# Supplementary material for: Inside the decentralised casino: A longitudinal study of actual cryptocurrency gambling transactions
Source: PLoS One. 2020 Oct 28;15(10):e0240693. doi: 10.1371/journal.pone.0240693 (PMC7592737; doi:10.1371/journal.pone.0240693)
Supplement: S1 Table — The behaviours of the top 5% and other 95% of casino bettors by total amount wagered, reprinted with no modifications from LaBrie et al’s 2008 study on casino game players [1], on which the present study’s methodology is based. It is included here following reviewers recommendations for comparison. (PDF) [file pone.0240693.s001.pdf]

**S1 Table.** Gambling behaviour of extreme 5 and 95% subgroups of casino bettors, reprinted from LaBrie et al's 2008 study [1].

| | Most involved casino bettors top 5% ($n = 212$) | | Other 95% of participants ($n = 4,010$) | |
|---|---|---|---|---|
| **Measure** | **Mean (SD)** | **Median** | **Mean (SD)** | **Median** |
| Age | 34 (9) | 32 | 30 (9) | 28 |
| Duration | 476 (232) | 529 | 290 (233) | 246 |
| Frequency | 24% (17) | 20% | 16% (21) | 7% |
| Number of bets | 24,558 (36,779) | 10,465 | 2,403 (7,819) | 486 |
| Bets per day | 285 (344) | 188 | 107 (176) | 46 |
| Euros per Bet | 213 (682) | 25 | 25 (97) | 4 |
| Total Wagered | 345,579 (354,890) | 233,195 | 10,338 (19,360) | 2,284 |
| Net Loss | 8,746 (11,213) | 6,698 | 422 (939) | 107 |
| Percent Loss | 2.6 (3) | 2.5 | 8.0 (12) | 5.9 |

1. LaBrie, R.A., Kaplan, S.A., LaPlante, D.A., Nelson, S.E., Shaffer, H.J.: Inside the virtual casino: A prospective longitudinal study of actual internet casino gambling. European journal of public health **18**(4), 410–416 (2008)
