# Supplementary material for: Inside the decentralised casino: A longitudinal study of actual cryptocurrency gambling transactions
Source: PLoS One. 2020 Oct 28;15(10):e0240693. doi: 10.1371/journal.pone.0240693 (PMC7592737; doi:10.1371/journal.pone.0240693)
Supplement: Attachment — Submitted filename: Review1 22jul20 PONE-D-20-20436.pdf [file pone.0240693.s002.pdf]

Manuscript Number: PONE-D-20-20436
Article Type: Research Article
Full Title: Inside the Decentralized Casino: A Longitudinal Study of Actual Cryptocurrency Gambling Transactions

Minor:

1. Improve language especially in abstract.
2. Include graphical diagrams to enhance understandability.
3. A comparative graph or table with existing analysis of similar type on similar apps have to be included in result portion.

Major: The statistics of data collected from application like Etheroll, and Dice2Win is not shown and the span over which data is collected.

1. The summary of data and the periods over which data has been collected require a graphical representation before the final analysis is presented.
2. The design of algorithm/method which conducted the analysis is not shown in any form (textual or graphical).
3. Analysis required to be interlinked with the suggested improvements and detected issues which can help improve the online gambling through games. The Author has to lead readers to an application analysis.
