# Supplementary material for: Inside the decentralised casino: A longitudinal study of actual cryptocurrency gambling transactions
Source: PLoS One. 2020 Oct 28;15(10):e0240693. doi: 10.1371/journal.pone.0240693 (PMC7592737; doi:10.1371/journal.pone.0240693)
Supplement: Attachment — Submitted filename: Response to Reviewers.pdf [file pone.0240693.s003.pdf]

PLOS One Response to Reviewers

We would like to thank the reviewers for their time, valuable suggestions, and constructive comments! We also believe the scholarly potential of this new area to be very exciting, and have made several improvements to address your comments. We specifically worked to improve the clarity of the paper and the analysis it presents.

Please find each of the issues raised below, followed by a summary of our improvements;

1. Improve language especially in abstract [R1]

We have improved the language throughout by removing complex phrases, simplifying sentence structures in all sections, and improving the overall readability of the paper throughout. All of our changes are highlighted in the tracked changes document for your convenience. Special attention has been given to the abstract which is now more accessible.

2. Include graphical diagrams to enhance understandability [R1]

Two new figures have been added to enhance understandability, the first visualises the periods over which the data have been collected, and the second shows summary statistics for the data collected in graphical form. These diagrams together bring much needed clarity to the scope of the data collection performed, and provide the reader with a more intuitive understanding of the scale of our investigation.

3. A comparative graph or table with existing analysis of similar type on similar apps have to be included in results portion [R1]

Given the youth of the technology used in these apps, no such analysis of similar type exists in the current literature so this is currently not possible. This considered, we have included a table from the study on which our methodology is based, which also targets online (though not decentralised) casino game players for comparison, and have added some comparative statements in the results portion to better frame our findings.

4. The statistics of data collected from applications like Etheroll, and Dice2Win is not shown and the span over which data is collected; The summary of data and the periods over which data has been collected require a graphical representation before the final analysis is presented. [R1]

Both diagrams added to address (2) add to better describing the data collected as part of this research. On top of this, a table has been added and referenced in the *Data Sample* section presenting a number of statistics to more comprehensively describe the data collected from these applications. This table combined with the new figures comprehensively describe the dataset, which will be available through an OSF repository linked throughout.

5. The design of algorithm/method which conducted the analysis is not shown in any form (textual or graphical) [R1]

To make our analysis clearer we have included pseudo-code of the bot-detection procedure, and have added sentences throughout highlighting our fully documented and open source code which is available on github. Our study is fully and quickly replicable using the library referenced, and we invite our readers to do so by providing full access to our data and code.

6. Analysis required to be interlinked with the suggested improvements and detected issues which can help improve the online gambling through games. The author has to lead researchers to an application analysis. [R1]
We have integrated the changes outlined above into a more consistent narrative, but must stress that this paper's aim is not to improve online gambling through games, but rather to improve the study of player behaviours using the new paradigm of cryptocurrency transaction data. We have clarified this in the introduction, emphasising that the application analysis this paper presents is the first step towards developing a better understanding of players, and therefore lays the foundation for more advanced analytical methods in this advanced and emerging domain. The youth and lack of existing work in this area is also stressed in the introduction, better leading the reader to the reason for our application analysis.

We hope our revisions fully address your feedback, and would like to thank all reviewers for their encouraging comments to improve our paper.
